# Immediate Effects of Distinct Intensities of Transcutaneous Spinal Direct Current Stimulation on Chronic Pain: A Randomized Controlled Trial

**DOI:** 10.3390/neurosci5040043

**Published:** 2024-12-02

**Authors:** Kariny Realino do Rosário Ferreira, Maria de Cássia Souza Macedo, Ana Luiza Guimarães Alves, Arthur Ferreira Esquírio, Bianca Rossi Botim, Gabrielly Souza Jacob, Mayra Evelise Cunha dos Santos, Gabriela Lopes Gama, Michelle Cristina Sales Almeida Barbosa, Alexandre Wesley Carvalho Barbosa

**Affiliations:** Laboratory of Non-Invasive Neuromodulation—LANN, Department of Physical Therapy, Federal University of Juiz de Fora, Av. Moacir Paleta 1167, São Pedro, Governador Valadares 36036-900, MG, Brazil; kariny.realino@estudante.ufjf.br (K.R.d.R.F.); mariacassia.macedo@estudante.ufjf.br (M.d.C.S.M.); ana.alves@estudante.ufjf.br (A.L.G.A.); arthurferreira.esquirio@estudante.ufjf.br (A.F.E.); bianca.botim@estudante.ufjf.br (B.R.B.); 11973445603@estudante.ufjf.br (G.S.J.); mayra.evelise@estudante.ufjf.br (M.E.C.d.S.); gabriela.gama@ufjf.br (G.L.G.); michellecsalmeida@ufjf.br (M.C.S.A.B.)

**Keywords:** transcranial direct current stimulation, electrotherapy, nervous system, spinal cord

## Abstract

This study aimed to assess the immediate effects of transcutaneous spinal direct current stimulation (tsDCS) on pain outcomes, measured using the visual analog scale (VAS) and pressure pain thresholds in a cohort of 55 participants experiencing chronic pain using a controlled, randomized trial with 55 participants allocated into 2 groups: 2 mA and 0.5 mA of tsDCS for 20 min. Anodal stimulation was applied on the 12th thoracic vertebra, with the cathode positioned on the 7th cervical vertebra. Pain outcomes were assessed before and post intervention using the VAS and pressure algometry. Between- and within-group differences, along with chi-square tests, were used to determine the differences and responsiveness. Significance was established at *p* < 0.05. Findings showed significant temporal effects for both VAS (*p* < 0.001) and pressure algometry (*p* = 0.04). However, no between-group differences were noted for the time × group factor for VAS (*p* = 0.46) and pressure algometry (*p* = 0.78). No significant between-group differences were observed for the responsiveness analysis. The results indicate that a single 20-min session of 2 and 0.5 mA tsDCS improves pain scores for both intensities equally. However, there were no statistically significant between-group differences in pain perception or pressure pain threshold.

## 1. Introduction

Pain is a complex phenomenon that goes beyond neural nociception. It is characterized by an unpleasant sensory and emotional experience associated with the actual or potential tissue damage [1]. The experience of pain results from complex brain interpretation, not limited to the nociceptive input [2]. Chronic pain is defined by persistent pain lasting more than 3 months, even after treatment onset [3]. The prevalence of chronic pain is ~20.5% of the US population [4]. In Brazil, the prevalence is approximately 45.3% [5]. Its etiology is multifaceted, arising from a spectrum of conditions including musculoskeletal injuries, chronic illnesses, and neuropathies [1,5].

Chronic pain is characterized by some features, including the intensity variability ranging from mild to severe and a usual oscillating nature, with periods of remission alternating with the symptoms’ occurrence [1,2,3,4,5,6]. Chronic pain disrupts the individuals’ quality of life, impairing their ability to engage in routine activities such as work, sleep, and social interactions [1,2,3,4,5,6,7]. Previous studies have indicated that psychological factors significantly influence the way people react to painful stimuli and that there is a direct relationship between socioeconomic level and the perception of pain intensity [8,9]. Many of these studies have highlighted family income as an explanatory variable, based on the principle that families struggling to meet their basic needs face a significant accumulation of stress [9]. Individuals trapped in this stress-pain-anxiety cycle tend to have a higher prevalence of chronic pain, experiencing greater intensity and frequency of pain [5,8,9].

Evidence indicates that chronic pain constitutes a significant burden worldwide, accounting for substantial health expenditures [10]. An analysis of the overall US healthcare expenditures in 2016 revealed that low back and neck pain incurred the highest costs, totaling US$ 134.5 billion [10]. Additionally, other musculoskeletal disorders represented the second-largest expense, amounting to US$ 129.8 billion [9,10,11]. Moreover, the management of chronic pain is still a challenge, often defying conventional therapeutic paradigms and demanding comprehensive, tailored interventions [12]. In this context, transcutaneous spinal direct current stimulation (tsDCS) emerges as a non-invasive method that shows promising effects on neurophysiological processing, particularly for chronic pain management [13,14].

The basic tsDCS principle involves applying an electrical current between two electrodes. An anodal electrode is often placed on the thoracic vertebrae (T10-T12), while the cathode is placed on a referenced area, such as the somatosensory cortex [13,15] or cervical vertebrae (C7). Low-intensity current flows through the skin, bones, cerebrospinal fluid, and nervous tissue [13,14]. Typically, a direct current of 1–2.5 milliamps (mA) is used [16]. Electrode positioning creates an electrical gradient that can alter the metabolic activity of underlying neural tissues [16,17].

tsDCS has demonstrated the ability to induce neurophysiological changes both locally at the site of the stimulation along the spine, as well as distally in supraspinal and even cortical regions [16,18]. Two non-mutually exclusive synaptic mechanisms of cathodic polarization have been postulated for tsDCS, ultimately resulting in the facilitation of spinal impulses. 1. Inhibition of the γ-aminobutyric acid (GABA)ergic system: GABA is the primary inhibitory neurotransmitter in the central nervous system (CNS), balancing excitatory signals and preventing neural overactivity. GABAergic neurons act by hyperpolarizing postsynaptic membranes, reducing the likelihood of action potentials and thus lowering neuron excitability. This inhibitory function is essential for maintaining stable neural circuits and preventing excitotoxicity. In the context of pain, GABAergic inhibition is crucial. Reduced GABAergic activity can lead to heightened excitability of pain-related pathways, contributing to chronic pain states. In contrast, increased GABAergic function generally correlates with analgesia, as it dampens nociceptive transmission. GABAergic inhibition is closely linked to neuroplasticity—the ability of the nervous system to adapt and reorganize. The inhibition of GABAergic activity can lead to a state known as disinhibition, which is a key mechanism for inducing plastic changes. Disinhibition temporarily reduces the threshold for synaptic changes, allowing the formation of new connections or the strengthening of existing ones. 2. The direct overexcitation of postsynaptic neurons, likely stemming from enhanced glutamate release at the spinal level [16,19]: Glutamate is the primary excitatory neurotransmitter in the CNS. It acts by binding to postsynaptic receptors, such as NMDA (N-methyl-D-aspartate) and AMPA (α-amino-3-hydroxy-5-methyl-4-isoxazolepropionic acid) receptors, which depolarize postsynaptic neurons and increase their likelihood of firing action potentials. Glutamate release at synapses is essential for processes such as synaptic plasticity, learning, and memory. In the spinal cord, glutamate plays a vital role in the transmission of nociceptive (pain-related) signals from the periphery to the CNS. By enhancing glutamate release, tsDCS can increase excitatory input to neurons involved in descending pain modulation pathways. This effect can override aberrant pain signaling often seen in chronic pain conditions, shifting the balance in spinal circuits toward inhibitory control. Enhanced excitability in these pathways may stimulate inhibitory interneurons, which work to reduce pain signaling to the brain. Recent evidence has confirmed a synaptic role from cathodic stimulation on spinal interneurons in humans [20].

Several tsDCS studies have demonstrated notable clinical effects on chronic pain perception, including a significant reduction in Visual Analogue Scale (VAS) scores, along with progressive improvements in quality of life and the alleviation of associated symptoms [13,17,21,22].

However, based on a literature review, no studies were found that examined the immediate effects of tsDCS in patients with chronic pain using distinct intensities of 0.5 mA and 2.0 mA. The aim of the current study was to determine whether there is a difference in the magnitude of pain perception as self-reported by the Visual Analog Scale (VAS) and measured using the pressure algometry. Therefore, the present study aimed to analyze the immediate pain responses to tsDCS application at different intensities (2.0 and 0.5 mA).

## 2. Materials and Methods

### 2.1. Participants

**Figure 1:**
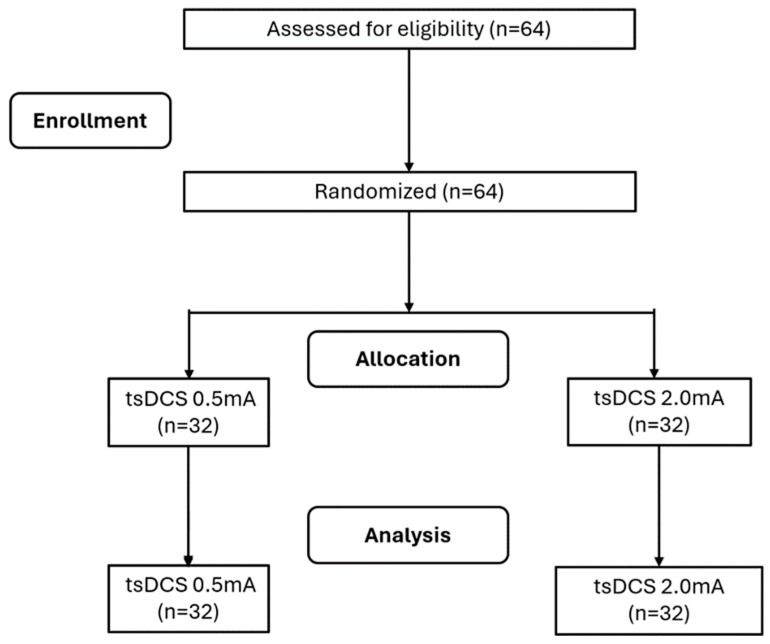
Flow diagram of the participants (2024). tsDCS: transcutaneous direct current stimulation.

Fifty-five individuals (18–85 years) from both sexes participated in the study. The sample calculation was performed using G-Power software [18,19]. The a priori two-tailed biserial model sample size calculation was performed using G-power software (version 3.1, Franz Faul, University of Kiel) considering an effect size of 0.602 obtained from a previous, similar study [22] using the within-group comparison (experimental group) with an alpha of 5% and a minimum sampling power (1 − β) of 95%. A sample size of 38 participants was returned with an actual power of 0.951. After a 40% drop-out, the final sample constituted 64 participants. The inclusion criteria inclided adults aged between 18 and 85 years old, with normal hearing, no history of neuropsychiatric disorders or self-reported use of psychoactive substances, and suffering from chronic pain (lasting more than 3 months). The exclusion criteria were the self-reported absences of the following conditions: cardiac pacemakers, pregnancy, injuries, and metal in or near the electrode position on the spine (e.g., aneurysm clips or coils, firearm projectile fragments). Participants were included or excluded according to the presented criteria and were divided randomly into 2 groups of 32 people each: the tsDCS 2 mA group and the tsDCS 0.5 mA group (Figure 1). The randomization sequence was performed using the http://www.randomizer.org website (accessed on 10 January 2023), considering 64 participants, an input of 2 groups, and the uniqueness of each position in the randomization ranking. The allocation concealment was preserved by informing the therapist of the participant’s group assignment only after their enrollment in the research. This randomized clinical trial was conducted in accordance with the Declaration of Helsinki and approved by the Ethics Committee of the Federal College of Juiz de Fora (number 69441023.5.0000.5147). Additionally, the study was registered in the Brazilian clinical trials registry (number RBR-9252kwm). The participants were briefed on the assessment and intervention protocols conducted throughout the study, and each one provided written consent after being fully informed about the procedures.

### 2.2. Transcutaneous Spinal Direct Current Stimulation (tsDCS)

**Figure 2:**
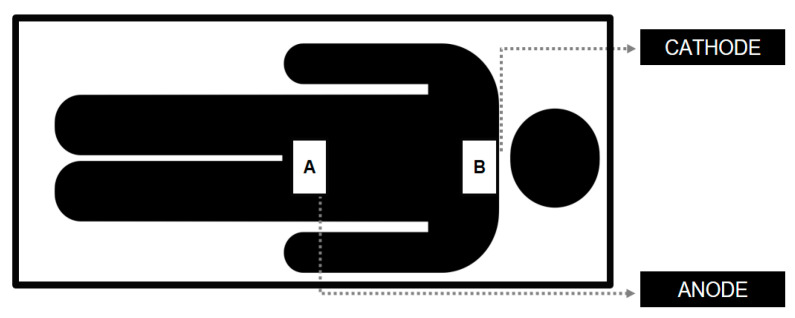
tsDCS electrodes positioning (2024). (A): 12th thoracic vertebra; (B): 7th cervical vertebra.

Electrical stimulation was applied using analog equipment (Neuroeletrical, São Paulo, Brazil) with a 4-mA maximum current. The applied current intensities were 2 mA or 0.5 mA, for 20 min. Two 50 cm^2^ (10 × 5 cm) rubber electrodes wrapped in a saline solution-soaked sponge were used. The anode was placed over the 12th thoracic vertebra, while the cathode was positioned over the 7th cervical vertebra (Figure 2). A 30-s ramp-up current initiated the procedure, and a 30-s ramp-down finished the stimulation period. The choice of electrode placement for tsDCS—anodal stimulation at T12 and cathodal stimulation at C7—was selected to maximize the modulation of spinal pathways associated with pain processing. This configuration has been shown to influence nociceptive pathways by facilitating the inhibition of pain-transmitting neurons and enhancing the excitability of descending inhibitory control systems [17]. Intensity settings of 0.5 mA and 2 mA allowed for comparison across a spectrum of stimulation strengths, with prior studies suggesting that even low-intensity stimulation can produce significant neuromodulatory effects on pain.

### 2.3. Experimental Protocol

The study collected pre- and post-experimental pain levels from each participant utilizing the Visual Analogue Scale (VAS) [23] to gauge subjective perception and pressure algometry to measure the pressure-pain threshold [24]. For the VAS, participants were seated and instructed to indicate their current pain level by marking a 10-cm line with a pen. Pressure algometry with a validated algometer (MED.DOR Ltd., Dores de Campos, Minas Gerais, Brazil; maximum compression = 50 kgf, precision = 0.1 kgf, 3-digit display) involved participants sitting in a chair with their feet flat on the floor, hands resting on their thighs, and their torso upright. A trained rater then performed three consecutive repetitions on the skin surface of the participant who had the most pronounced self-reported pain [24]. The location indicated by the participant received progressive pressure of 1 kg/s controlled by a metronome until the participant felt pain, which in turn was indicated by raising their hand. To avoid any subconscious rater bias, the LCD display was kept turned away from the rater’s gaze. Once the procedure was completed, the values were recorded.

### 2.4. Data Extraction

For the VAS, the raters measured the vertical marking using a caliper and subsequently stored it in an online Microsoft Excel spreadsheet. For algometry, the data were immediately extracted and organized into digital format using an online Microsoft Excel spreadsheet. For responsiveness, the data were categorized into responsive (1) and non-responsive (2).

### 2.5. Statistical Analysis

The Shapiro-Wilk and the Levene tests were used to assess data normality and homogeneity, respectively. After a logarithmic transformation, the Shapiro-Wilk test was performed to assess the sample’s normality. Intention-to-treat analysis was performed using a factorial ANOVA with repeated measures to assess between- and within-group differences considering the time and time × group interactions. The effect size was calculated using the partial eta square (η^2^_p_). The magnitude of the η^2^_p_ was qualitatively interpreted using the following thresholds: ~0.01 (small), ~0.06 (moderate), and ~0.14 (large). The chi-square test for association was used to assess the responsiveness frequencies. Significance was established at *p* < 0.05. All assessments were conducted using the Jamovi software (Jamovi project, version 0.9 2020).

## 3. Results

The participants’ characteristics are shown in Table 1. The findings (Table 2) revealed a significant temporal effect for both VAS (F = 21.057; *p* < 0.001; η^2^_p_ = 0.284 [large]) and pressure algometry (F = 4.430; *p* = 0.04; η^2^_p_ = 0.07 [moderate]). However, there were no significant between-group differences for the time × group interaction for VAS (F = 0.539; *p* = 0.466) nor for the pressure algometry (F = 0.07; *p* = 0.78). No significant differences were observed for between-group frequencies (χ^2^ = 2.2; *p* = 0.33).

## 4. Discussion

The present findings showed immediate differences in terms of pain perception and pressure pain thresholds after both 2 and 0.5 mA intensities, with respective large and moderate effect sizes. However, no between-group (2 mA vs. 0.5 mA) differences were observed after tsDCS.

Evidence regarding tsDCS suggests that spinal neurophysiological effects might follow a non-linear dose-response curve, where intensities beyond a certain threshold do not proportionally increase the effect [14]. The dose ceiling concept is supported by studies where higher intensities (e.g., 2.5 mA) did not produce significantly greater analgesic effects than moderate intensities (e.g., 0.5–2.0 mA). This might imply that tsDCS, even during low intensities, could achieve the near-maximal stimulation of relevant pathways or pain modulation processes, suggesting an optimal intensity threshold. Studies have proposed that spinal neuroplasticity may be maximally responsive to moderate current levels, as higher currents could lead to saturation or even paradoxical effects on neuron excitability [14,18]. This saturation may indicate an optimal stimulation window, above which additional current does not yield increased analgesic benefits and could disrupt homeostatic neuroplastic mechanisms.

Chronic pain conditions often involve central sensitization, where spinal neurons become hypersensitive due to persistent excitatory signaling [1,2,3]. By using tsDCS to target specific spinal pathways, the increased glutamate release and resulting overexcitation can help recalibrate the excitatory-inhibitory balance in the spinal cord, potentially reducing hyperalgesia [14,16]. Overexcitation from increased glutamate release may facilitate long-term potentiation (LTP), a form of synaptic plasticity where repeated activation strengthens synaptic connections. This would be beneficial for chronic pain treatment, as LTP in descending pain inhibition pathways can create lasting changes that counteract pain hypersensitivity. While glutamate-induced overexcitation is useful for therapeutic modulation, excessive glutamate can lead to excitotoxicity, a damaging process where prolonged glutamate activity harms neurons. Therefore, controlled intensities in tsDCS are crucial to safely promote excitatory effects without overwhelming neuronal circuits.

The current findings aligned with those of Guidetti et al. (2021), who implemented tsDCS for chronic pain participants. However, some differences from the current study must be addressed [17]. The T12 anodal and C7 cathodal positioning in the present study contrasts with the previous study, which chose the T12 anodal and somatosensory area cathodal montage. The current study also employed 2.0 mA intensity, while Guidetti et al. (2021) applied 2.5 mA. Additionally, a 0.5 mA comparison group was included in the present set-up, whereas the previous study employed a sham therapy so the current would not exert any significant influence on the assessed outcomes. However, in another study by Guidetti et al. (2023), which delved into the modeling of electrical fields in tsDCS, it was observed that the relationship between the electrical dose and clinical response remains unclear in tsDCS, requiring further investigation [14]. A non-linear pattern is often expected considering the current intensity and the physiological outcome, suggesting that higher field intensities do not inherently correlate with increased effects. The present study had similar results to the 0.5 mA dose, which yielded similar outcomes to 2 mA.

Notably, Guidetti et al. (2021) observed significant pain score differences after one week of intervention [17]. However, their findings were based on a small sample size. Regarding the sample characteristics, a robust sample size calculation was carried out for the present study, with a diverse set of participants considering diagnosis and age in comparison with other studies.

Choi et al.’s (2019) pilot investigation examined the immediate response to chronic neuropathic pain following spinal cord injury through tsDCS application with T12 anodal and CZ cathodal positioning [22]. The assessment was performed immediately following the current, with additional 1-h and 2-h post-session assessments. Despite no differences being immediately noted, significant differences were not observed; the post hoc analysis revealed a significant difference from baseline at the 1-h mark. This finding potentially supports the hypothesis that tsDCS effects occur cumulatively over an extended period of treatment.

The findings reported by Lenoir et al. (2018), who examined the effects of tsDCS in eight healthy female participants, indicated that tsDCS attenuates the nociceptive stimuli and significantly influences the LEP-N2 wave elicited by nociceptive laser stimulation applied to the lower limbs [16]. The authors used a temperature-controlled CO_2_ laser to activate Aδ and C fiber thermonociceptors, providing brief pulses of radiant heat to the hands and feet with pre-established settings for activation thresholds of Aδ and C fiber thermonociceptors [16]. The authors proposed that the observed selective neuromodulatory response may be plausibly attributed to the anodal blockade of axonal conduction within the spinal cord. Furthermore, it was suggested that the post-stimulation effects of low thoracic tsDCS could be associated with the synaptic modulation of local processing and/or transmission of nociceptive stimuli at the dorsal horn level.

Another explanation lies in the neurophysiological underpinnings of chronic pain, which involve complex spinal and cortical circuits where GABAergic and glutamatergic systems play crucial roles. The present study’s intensities could similarly affect these pathways, particularly if cathodal stimulation reduces inhibitory GABAergic activity or enhances excitatory neurotransmission via glutamate. A previous study conducted by Bocci et al. (2015) proposed that tsDCS modulates inhibitory GABA(A)ergic drive, as evidenced in a small sample of 10 healthy participants [18]. Similarly, the authors opted for cathodal stimulation on the shoulder and anodal stimulation at T11, administering an intensity of 2.5 mA for 20 min in a single session. Despite the absence of immediate effects, electrophysiological assessment revealed some molecular-level alterations. Nonetheless, caution is suggested when interpreting those effects, mainly due to sample size limitations and the absence of sample size calculation.

The current study has some limitations that must be addressed. The sample’s heterogeneity concerns the diagnosis and age, as well as the heterogeneity of pain duration. However, the results followed the same pattern for all participants, suggesting that such heterogeneity was not a cofactor for which to change the current analysis. Thus, the chronic pain seemed to drive the observed effects regardless of diagnosis or age. Further research is required to clarify tsDCS dynamics in chronic pain in short- and long-term protocols featuring an increased number of sessions. Due to equipment constraints, which did not allow for pre-programmed intensity, the present design was not double-blinded. Another limitation was the lack of a sham group. Thus, a placebo effect could also have occurred; other studies have acknowledged the 2 mA effect for tsDCS. As distinct intensities have shown similar effects, it is advisable to integrate a sham therapy group for further studies.

Throughout the study, participants experienced some adverse effects, including skin burns at the electrode application site (impacting ~20% of the sample) and headaches during the session. These effects, as previously documented in the literature [25], were mitigated by diluting the conductive saline fluid in distilled water, yielding a 0.6% solution instead of 0.9%.

During the study, nine participants were lost: four chose not to undergo immediate reassessment, and five felt discomfort in the posture selected for the application. However, their baseline assessments were included in the statistical procedure to preserve the intention-to-treat analysis.

From a clinical perspective, the current study’s findings underscore the viability of tsDCS as a complementary treatment for chronic pain. While pharmacological treatments remain a cornerstone of pain management, the integration of non-invasive neuromodulatory techniques, such as tsDCS, could offer a safe, cost-effective approach that minimizes the side effects associated with long-term medication use. Future research should explore the cumulative effects of repeated tsDCS sessions, as chronic pain management often requires sustained intervention to achieve lasting benefits.

## 5. Conclusions

The present results suggest that a single 20-min session of 2 and 0.5 mA tsDCS improves chronic pain perception equally for both intensities. The study provides valuable insights into the immediate effects of tsDCS on chronic pain, suggesting that low-intensity stimulation is sufficient to achieve meaningful pain relief without necessitating higher intensities. This finding supports the potential for tsDCS to become an accessible treatment modality, especially given its portability and ease of use in various clinical settings. Further exploration of the optimal parameters for tsDCS application, including frequency, duration, and electrode placement, could lead to improved pain management strategies and broader implementation in clinical practice.

## Figures and Tables

**Table 1:** Participant characteristics.

Characteristics	tsDCS 2.0 mA Median (Min–Max)	tsDCS 0.5 mA Median (Min–Max)	*p*-Value
Age (years)	46.5 (21–84)	52 (20–80)	0.54
Weight (kg)	71.5 (51–45)	72 (51–45)	0.06
Height (cm)	165 (140–194)	165 (151–176)	0.41

**Table 2:** Visual analogue scale (VAS) and pressure-pain threshold (PPT) results.

Group	VAS_PRE_ Mean (SD)	VAS_POST_ Mean (SD)	PPT_PRE_ Mean (SD)	PPT_POST_ Mean (SD)
tsDCS 2 mA	4.83 (2.74)	3.68 (2.55)	2.31 (1.46)	2.56 (1.56)
tsDCS 0.5 mA	4.79 (2.17)	3.97 (2.47)	2.56 (1.48)	2.88 (1.82)

## Data Availability

The data from this study will be made available upon request.
